# Infection risks in multiple myeloma: a systematic review and meta-analysis of randomized trials from 2015 to 2019

**DOI:** 10.1186/s12885-021-08451-x

**Published:** 2021-06-26

**Authors:** Nicole Balmaceda, Muhammad Aziz, Viveksandeep Thoguluva Chandrasekar, Brian McClune, Suman Kambhampati, Leyla Shune, Al-Ola Abdallah, Faiz Anwer, Aneela Majeed, Muzaffar Qazilbash, Siddhartha Ganguly, Joseph McGuirk, Ghulam Rehman Mohyuddin

**Affiliations:** 1 University of Kansas Cancer Center, 2650 Shawnee Mission Pkwy, Westwood, KS 66205 USA; 2 University of Toledo, 3000 Arlington Ave, Toledo, OH 43614 USA; 3 Mayo Clinic Arizona, 5777 E Mayo Blvd, Phoenix, AZ USA; 4 University of Utah, 50 N Medical Dr., Salt Lake City, UT 84132 USA; 5 U.S. Department of Veterans Affairs, Kansas City Medical Center, 4801 Linwood Blvd., Kansas City, MO 64128 USA; 6 Cleveland Clinic, 9500 Euclid Ave, Cleveland, OH 44195 USA; 7 University of Texas MD Anderson Cancer Center, Houston, Texas, 1515 Holcombe Blvd., Houston, TX 77030 USA

**Keywords:** Multiple myeloma, Proteasome inhibitors, Anti-CD38, Cytotoxic therapy, Infection

## Abstract

**Background:**

Patients with multiple myeloma (MM) remain at an increased risk of infection due to the disease process, as well as the ensuing treatments.

**Methods:**

We performed a systematic review to evaluate the monthly risk of grade III/IV infection, pneumonia, and neutropenia in patients with myeloma enrolled in randomized clinical trials (RCTs).

**Results:**

The risk of grade III or higher infection, pneumonia, and neutropenia persists among all phases of treatment. There was no statistical difference in grade III or higher infection, pneumonia, and neutropenia between frontline and relapsed/refractory setting. In the maintenance setting, the complications of infection, pneumonia, and neutropenia were low, but not negligible. Three-drug regimens were no more likely than two-drug regimens to have an increased risk of Grade III or higher infection.

**Conclusions:**

This is the first study to quantify the monthly risk of grade III or higher infection, pneumonia, and neutropenia across different treatment regimens in the frontline, maintenance, and relapsed/refractory settings. The results of our systematic review demonstrate a significant risk for severe infection, pneumonia, and neutropenia in patients with MM. Further studies are needed to determine the value of antibiotic prophylaxis in a broader myeloma patient population, as well as other approaches that will further mitigate the morbidity and mortality related to infection in this vulnerable patient population.

**Supplementary Information:**

The online version contains supplementary material available at 10.1186/s12885-021-08451-x.

## Introduction

Patients with multiple myeloma (MM) remain at an increased risk of infection due to the immunosuppressive nature of the underlying disease process, as well as the ensuing treatments [1–3]. Postulated risk factors for infection risk in myeloma include impaired host defenses with disease progression (leukopenia, T-cell immunodeficiency, hypogammaglobulinemia, poor performance status, increasing age, renal failure), multifactorial immunosuppression with prolonged steroid exposure and previous treatments (reduced CD4+, CD45+, CD19+, and NK cells), and disease evolution with mutational changes and clonal evolution and heterogeneity [4]. The risk of infection is the greatest within the first 3 months following diagnosis, and infections remain an important contributing factor to early morbidity and mortality for patients with MM [2, 5, 6].

Regimens known to increase the risk of severe infections include immunomodulatory drugs (IMiDs) and proteasome inhibitors (PIs). IMiDs like lenalidomide and pomalidomide, cause neutropenia, increasing risk of infection [7, 8]. The PI, bortezomib is associated with reactivation of varicella-zoster-virus due to impairment of T-cell function [9].

Given the increased risks of severe morbidity and mortality, it is imperative to assess the degree of immunosuppression and risk of infection with different treatment regimens, across all phases of treatment. Such information is useful for patients and providers in the risk assessment and mitigation decision-making process. No study has every reported the monthly-associated risk of infection with different treatment regimens in clinical trials across frontline, maintenance, and relapsed/refractory settings. We performed a systematic review and meta-analysis evaluating the monthly risk of infection, pneumonia, and neutropenia in patients with myeloma on treatment enrolled in randomized clinical trials (RCTs).

## Methods

### Search strategy

Three databases were searched i.e., MEDLINE/PubMed, Embase, and Cochrane Registry of Controlled Trials. An example search strategy using Embase is highlighted in Supplementary Table 1. Two independent reviewers (GRM, NB) screened all studies, and any conflict was resolved through mutual discussion. Furthermore, for the purpose of our analysis, we strictly adhered to predefined reporting criteria. This systematic review and meta-analysis were performed according to the Preferred Reporting Items for Systematic Reviews and Meta-Analyses (PRISMA) recommendations [10].

**Table 1:** Characteristics of randomized clinical trials included in study

Author (Year)	Trial Name	Study Phase	Phase Treatment	Regimen	No. Patients	Median Age (Years)	Median Duration of Treatment (Months)
Niesvizky (2015) [14]	UPFRONT	3	ND	Bortezomib/dexamethasone	165	74.5	6
				Bortezomib/thalidomide/dexamethasone	158	73	4.6
				Bortezomib/melphalan/prednisone	163	72	4.7
Richardson (2015) [33]		2	RR	Elotuzumab/lenalidomide/dexamethasone	73	62	19.1
Mateos (2019) [15]	ALCYONE	3	ND	Daratumumab/bortezomib/melphalan/prednisone	346	71	13.5
Voorhees (2019) [16]	GRIFFIN	2	ND	Daratumumab/lenalidomide/bortezomib/dexamethasone	99	59	22.1
Usmani (2019) [38]	KEYNOTE-185	3	RR	Pembrolizumab/lenalidomide/dexamethasone	149	74	4.4
Mateos (2019) [40]	COLUMBA	3	RR	Subcutaneous daratumumab	260	65	7.5
				IV daratumumab	258	68	7.5
Spicka (2019) [34]	ADMYRE	3	RR	Plitidepsin/dexamethasone	167	64	3
Rosinol (2019) [17]	PETHEMA/GEM2012	3	ND	Bortezomib/lenalidomide/dexamethasone prior to transplant	458	58	6
Attal (2019) [35]	ICARIA-MM	3	RR	Isatuximab/pomalidomide/dexamethasone	152	68	10.2
Morgan (2019) [41]	TOURMALINE-MM3 study	3	M	Ixazomib maintenance	395	58	15.2
Moreau (2019) [36]	BELLINI	3	RR	Venetoclax/bortezomib/dexamethasone	194	66	18.7
Moreau (2019) [18]	CASSIOPEIA	3	ND	Daratumumab/bortezomib/melphalan/prednisone prior to and following transplant	536	59	8.9
Richardson (2019) [27]	OPTISIMISMM	3	RR	Bortezomib/pomalidomide/dexamethasone	278	67	8.8
Mateos (2019) [26]	KEYNOTE-183	3	RR	Pembrolizumab/pomalidomide/dexamethasone	120	65	4.1
Dimopoulos (2018) [30]	POLLUX	3	RR	Daratumumab/lenalidomide/dexamethasone	283	65	24.5
Jackson (2019) [24]	Myeloma XI	3	ND	Cyclophosphamide/bortezomib/dexamethasone induction	275	66	2.8
Horvath (2019) [19]	VCAT	3	ND	Bortezomib/thalidomide/prednisolone consolidation	103	58	10.2
Facon (2019) [23]	CLARION	3	ND	Carfilzomib/melphalan/prednisone	474	72	13.1
Facon (2019) [20]	MAIA	3	ND	Daratumumab/lenalidomide/dexamethasone	364	73	25.3
Lonial (2015) [25]	ELOQUENT-2	3	RR	Elotuzumab/lenalidomide/dexamethasone	318	67	17
Dimopoulos (2016) [37]	ENDEAVOR	3	RR	Carfilzomib	463	65	10
Spencer (2018) [31]	CASTOR	3	RR	Daratumumab/bortezomib/dexamethasone	243	64	13.4
Moreau (2018) [28]	A.R.R.O.W.	3	RR	Once weekly carfilzomib	238	66	9.5
				Twice weekly carfilzomib	235	66	7.3
Dimopoulos (2018) [29]	ELOQUENT-3	2	RR	Elotuzumab/pomalidomide/dexamethasone	60	69	8.4
Hajek (2016) [32]	FOCUS	3	RR	Carfilzomib	157	63	4.1
Durie (2017) [21]	SWOG S0777	3	ND	Bortezomib/lenalidomide/dexamethasone non-transplant	242	63	5.6
Bringhen (2019) [42]	EMN01	3	M	Lenalidomide maintenance	204	73	32.4
				Lenalidomide/prednisone maintenance	198	73	29.8
Zweegman (2016) [43]	HOVON-NSMG	3	M	Thalidomide maintenance	121	72	5
				Lenalidomide maintenance	124	73	17
Moreau (2016) [39]		3	RR	Ixazomib/lenalidomide/dexamethasone	361	66	15.9
Jacobus (2016) [22]	E1A05	3	NF	Bortezomib/lenalidomide/dexamethasone consolidation,	23		4.55
Gay (2015) [44]		3	M	Lenalidomide maintenance	117	57	28.9
				lenalidomide/prednisone maintenance	106	56	25.3

### Inclusion and exclusion criteria

Our search strategy was performed to include RCTs from January 1, 2015 to December 30, 2019. The search was last updated on April 1, 2020. Studies were only included for quantitative analysis if authors clearly reported the median duration of treatment or median number of cycles corresponding to their reported toxicities. If a study only reported combined leukopenia/neutropenia as a composite outcome, that study was not included in our neutropenia category. The use of antimicrobial prophylaxis was also obtained. Studies evaluating different phases of treatment (induction/consolidation/maintenance) that did not clearly elucidate reported timeframe of toxicities were not included in our analysis. All other studies including editorials, case reports, case series, review articles, case control, retrospective/prospective cohort, and single arm studies were excluded. Studies of regimens that only reported the efficacy/safety of autologous transplant were also excluded as our main focus was to evaluate the toxicity of MM regimens, including those used prior to or after a transplant, rather than the toxicity of the transplant itself. The search strategy was not restricted to language. Abstracts from conference proceedings that were captured via our search strategy (such as those listed on Embase) were also included.

### Data collection

Two authors (GRM and NB) performed and verified all data extraction. Extracted data was tabulated using Microsoft Excel (Microsoft, Redmond, Washington, United States). We identified number of participants in each study and characteristics of studies such as the nature of MM patient population (“transplant-eligible” versus “non-transplant-eligible”) and regimens used as “first-line” or “relapsed/refractory.” We also identified what class of drug was used in each regimen based on whether a PI/IMiD/Anti-CD38 agent was included or not. When pooled analysis was presented for a class of drugs, maintenance studies were excluded as the toxicities would be expected to be much lower. We also collected the publication year of data related to each study. We systematically screened each of the trials for outcomes pertaining to the incidence of infection, the grade/type of such infection, and neutropenia. Other infection-specific variables were captured including if antibiotic prophylaxis was permitted, number of participants who used prophylactic antibiotics, and death from infection. Immunomodulatory drugs were classified as thalidomide and its analogs (pomalidomide, lenalidomide). Bortezomib, carfilzomib, and ixazomib were identified as proteasome inhibitors. The median number of cycles received for each treatment regimen, in order to standardize outcome reporting was also collected.

### Primary and secondary outcomes

Our study had three primary outcomes which we assessed across each treatment phase of myeloma (frontline treatment, relapsed/refractory setting, and maintenance). The primary outcomes of the studies were the incidence of Grade III or higher infections per month on treatment amongst patients with MM enrolled in RCTs, the incidence of Grade III or higher pneumonia per month on treatment amongst patients with MM enrolled in RCTs, and the incidence of Grade III or higher neutropenia per month in treatment amongst patients with MM enrolled on RCTs.

### Heterogeneity and Bias assessment

We assessed heterogeneity in studies using the I^2^ statistic as defined by Cochrane Handbook for Systematic Reviews. I^2^ < 30%, 30–60%, 61–75, and > 75% were suggestive of low, moderate, substantial, and considerable heterogeneity, respectively [11, 12]. Study quality using Cochrane risk-of-bias tools for RCTs was assessed [11, 13]. The influence of individual studies was examined by leaving out one study and recalculating the meta-analysis.

### Statistical analysis

Pooled proportion rates for all outcomes were compared using risk ratio (RR) and 95% confidence intervals (CI) with *p*-values generated. A p-value of < 0.05 was considered statistically significant. We calculated outcomes using the DerSimonian-Laird method along with random effects. Due to software limitations, we multiplied the actual incidence/prevalence of outcomes by 100, to generate infection risk per 100 months. Results were displayed as per monthly risk of grade III or higher infection/pneumonia/neutropenia. Open meta-analyst (CEBM, Brown University, Rhode Island, USA) and Comprehensive Meta-analysis (Biostat, Englewood, New Jersey, US) were used as the computing software.

## Results

**Fig. 1:**
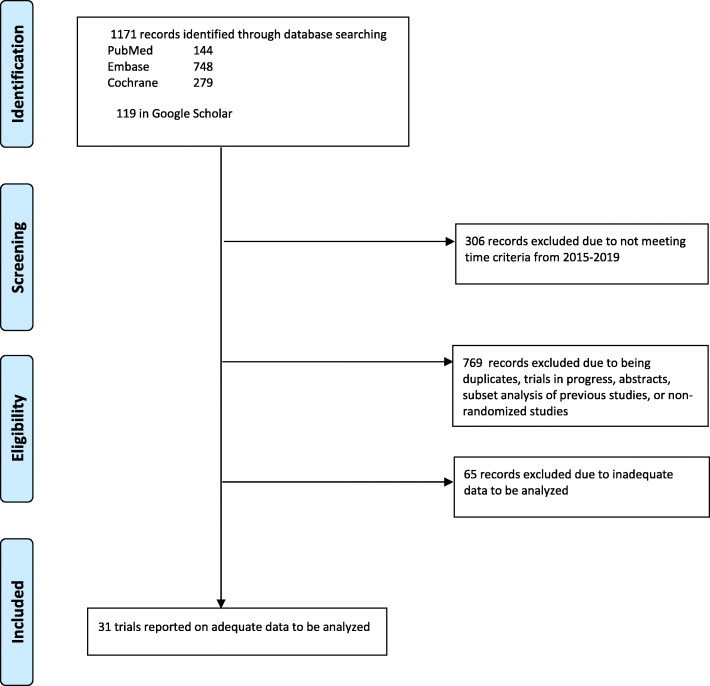
Flow diagram depicting our search strategy and study inclusion

After excluding trials not meeting defined time-period, duplicates, trials in progress with no results, and non-randomized studies, we included 31 RCTs for analysis in our study (Fig. 1).

Thirty-one studies clearly reported either incidence of Grade III or higher infection, pneumonia, or neutropenia, and provided a clear duration of treatment/number of cycles. Supplementary Table 2 lists characteristics of the studies included in our analysis. Supplementary Table 3 highlights the risk of bias in each of the included studies. The treatment regimens incorporated in these RCTs were analysed according to the phase of treatment (frontline, relapsed/refractory, and maintenance).

### Incidence of grade III or higher infection, pneumonia, neutropenia in RCTs evaluating patients in the frontline setting

**Fig. 2:**
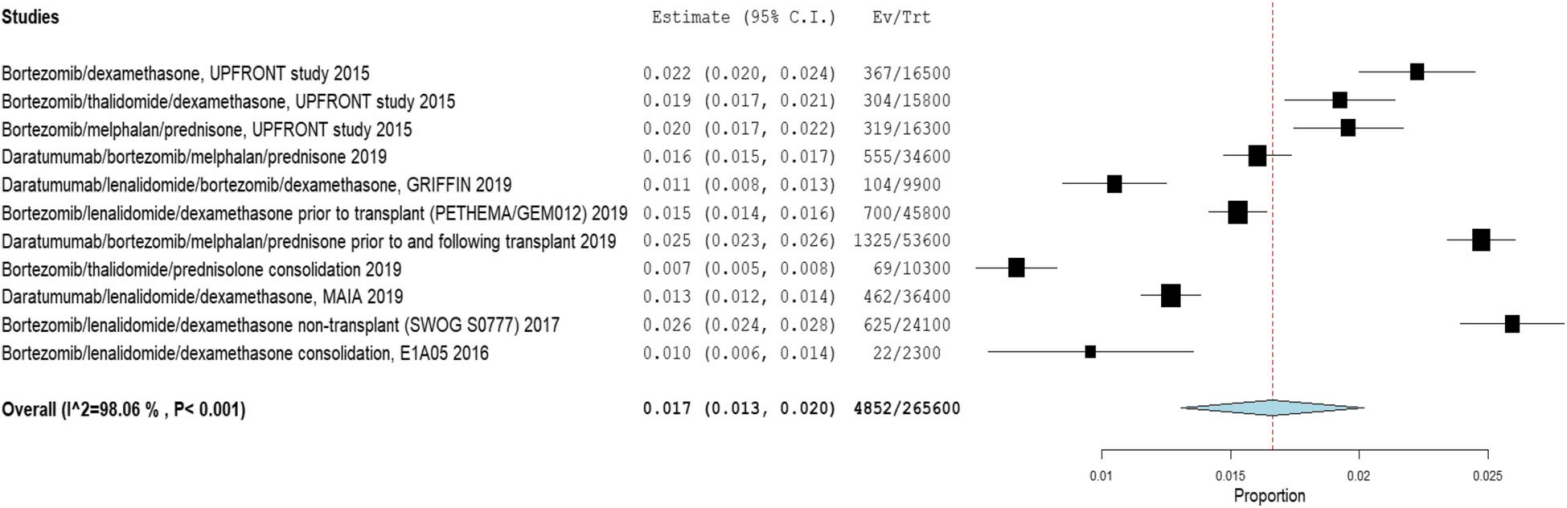
Incidence of Grade III or higher infection in frontline myeloma trials

Nine RCTs using 11 unique treatment regimens (*n* = 2656 patients) reported on the incidence of infection per cycle/month of therapy in the frontline setting (Fig. 2) [14–22].

In the frontline setting, bortezomib/lenalidomide/dexamethasone (VRD) was associated with a monthly incidence of grade III or higher infection of 2.6% (2.4–2.8%, I2 = 98.1%) [21] in non-transplant patients and 1.5% (1.4–1.6%, I2 = 98.1%) in transplant eligible patients [17]. Daratumumab/lenalidomide/dexamethasone (DRd) was associated with a monthly incidence of grade III or higher infection of 1.3% (1.2–1.4%, I2 = 98.1%) [20]. Bortezomib/dexamethasone (Vd) was associated with a monthly incidence of grade III or higher infection of 2.2% (2.0–2.4%, I2 = 98.1%) [14].

A total of 6 RCTs, 8 regimens, (*n* = 2231 patients) reported on the incidence of Grade III or higher pneumonia (Supplementary Figure 1) [14, 15, 17, 19, 20, 23]. Amongst these regimens, daratumumab/bortezomib/melphalan/prednisone was associated with the highest risk of monthly grade III or higher pneumonia in newly diagnosed MM (3.7% (3.5–3.9%, I2 = 99.4%) [15].

Ten RCTs with 12 regimens (*n* = 3459 patients) in newly diagnosed MM reported incidence of grade III or higher neutropenia per cycle/month of treatment (Supplementary Figure 2) [14–20, 23–25]. Bortezomib/melphalan/prednisone was associated with the highest risk of monthly grade III or higher neutropenia in newly diagnosed MM (4.0% (3.7–4.3%, I2 = 99.6%) [14]. Bortezomib/melphalan/prednisone had a significantly higher risk of monthly neutropenia compared to the other regimens in the UPFRONT study – bortezomib/dexamethasone [0.3% (0.2–0.4%, I2 = 99.6%)] and bortezomib/thalidomide/dexamethasone [0.5% (0.4–0.7%, I2 = 99.6%) [14]. VRD was associated with a monthly incidence of grade III or higher neutropenia of 2.1% (2.0–2.3%, I2 = 99.6%) [17].

### Risk of infection, pneumonia, neutropenia in RCTs evaluating patients in relapsed/refractory setting

**Fig. 3:**
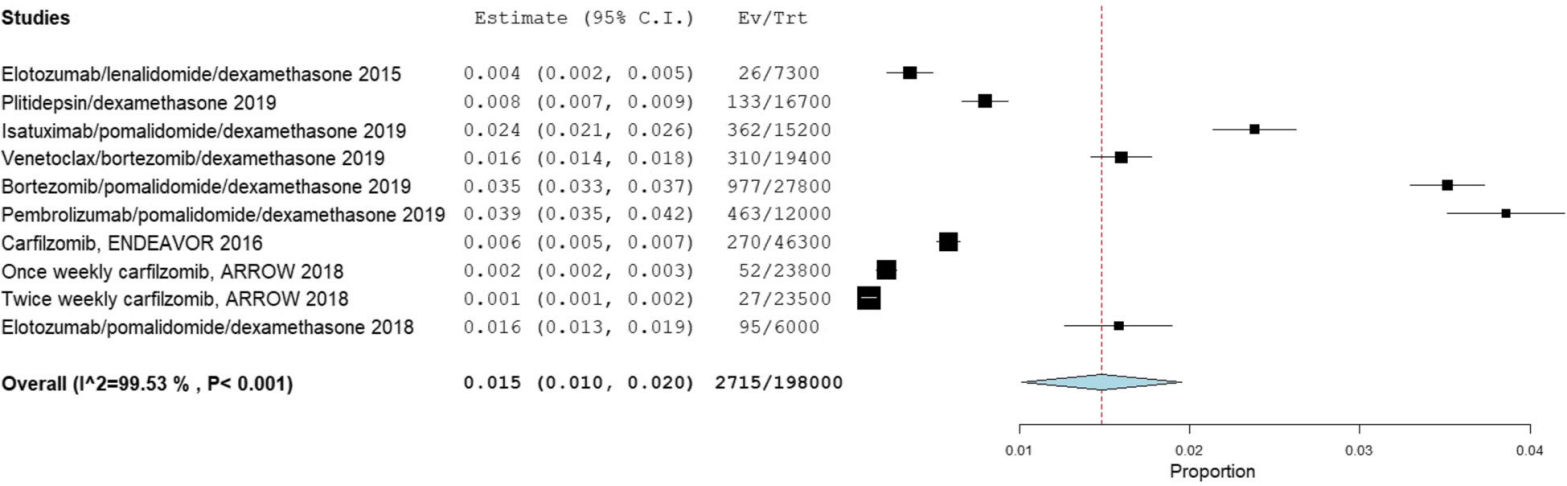
Incidence of Grade III or higher infection per month in relapsed/refractory myeloma trials

In the relapsed/refractory MM (RRMM) setting, we identified 9 RCTs with 10 treatment regimens (*n* = 1980 patients) reporting the incidence of Grade III or higher infection per cycle/month of treatment (Fig. 3, 26–34].

The highest monthly risk of infection was in patients treated with pomalidomide/dexamethasone-containing regimens: pembrolizumab/pomalidomide/dexamethasone, with a monthly risk for grade III or higher infection of 3.9% (3.5–4.2%, I2 = 99.5%) [26] followed by bortezomib/pomalidomide/dexamethasone at 3.5% (3.3–3.7%, I2 = 99.5%) [27].

Seven RCTs with 8 regimens (*n* = 1614 patients) reported the incidence of Grade III or higher pneumonia per cycle/month of treatment (Supplementary Figure 3) [26–32]. In the RRMM setting, the highest monthly risk of grade III or higher pneumonia was pembrolizumab/pomalidomide/dexamethasone [3.2% (2.9–3.6%, I2 = 97.5%)], whereas commonly used daratumumab-based regimens like DRd and Daratumumab/bortezomib/dexamethasone (DVd) were associated with monthly risks of Grade III or higher pneumonia of 0.8% (0.7–0.9%, I2 = 97.5%) [30] and 0.7% (0.6–0.8%, I2 = 97.5%) [31], respectively.

Fifteen RCTs, 17 regimens (*n* = 3691 patients) with RRMM reported the incidence of Grade III or higher neutropenia per cycle/month of treatment (Supplementary Figure 4) [26–40]. The monthly incidence of grade III or higher neutropenia with a contemporary pomalidomide-based triplet such as isatuximab/pomalidomide/dexamethasone was 8.3% (7.9–8.8%, I2 = 99.6%)] [35]. Commonly used daratumumab regimens such as DRd and DVd were associated with monthly risks of Grade III or higher neutropenia of 2.2% (2.0–2.4%, I2 = 99.6%) [30] and 1.1% (0.9–1.1%, I2 = 99.8%) [31], respectively.

### Incidence of infection, pneumonia, neutropenia in RCTs evaluating patients in maintenance setting

**Fig. 4:**
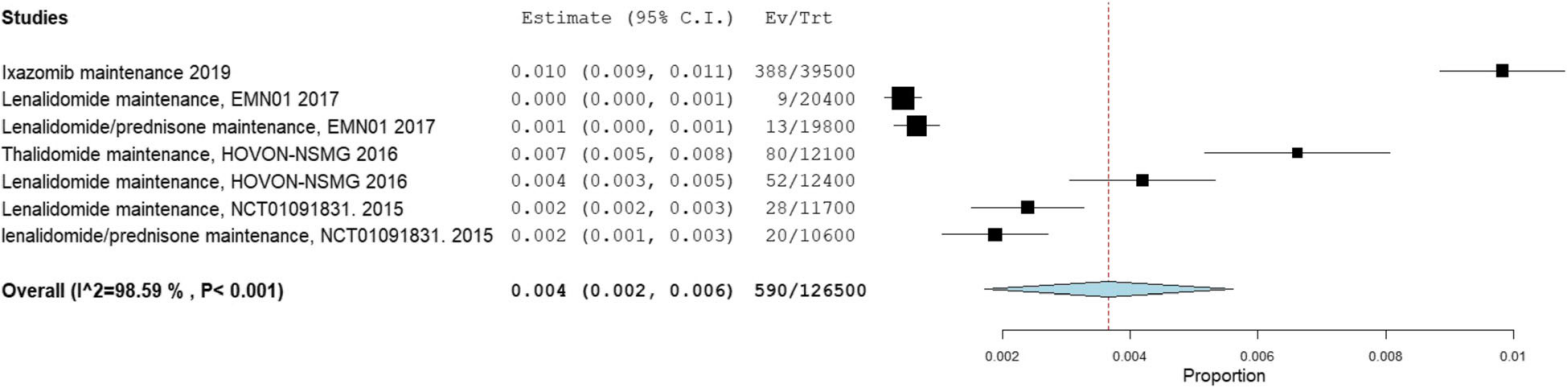
Incidence of Grade III or higher infection per month in myeloma maintenance trials

Four RCTs involving 7 regimens with 1265 patients on maintenance therapy reported incidence of Grade III or higher infection risk per month of treatment [41–44]. Across these 7 regimens the incidence of Grade III or higher infection risk per month of treatment was 0.4, 95% CI = 0.2–0.6% (Fig. 4).

Only one trial reported on incidence of grade III or higher pneumonia [42]. Four RCTs, with 7 treatment regimens involving 1265 patients with RRMM reported incidence of Grade III or higher neutropenia risk per month/cycle of treatment (Supplementary Figure 5) [41–44]. Across these 7 regimens, the incidence of Grade III or higher neutropenia per month was 0.5% (95% CI 0.4–0.7%).

### Comparison of risks for frontline versus relapsed-refractory patients

In the frontline setting, risk of grade III or higher infection, pneumonia, and neutropenia per month of treatment were 1.7 (95% CI = 1.3–2.0%), 1.3 (95% CI = 0.8–1.8%), and 1.9% (95% CI = 1.3–2.6%), respectively. In the RRMM groups, rates of Grade III or higher infection, pneumonia, and neutropenia per month were 1.5 (95% CI = 1.0–2.0%), 1.2 (95% CI = 0.9–1.6%), and 2.7% (95% CI = 2.1–3.3%), respectively.

### Comparison of risks for 2-drug versus 3-drug regimens

There was no statistical difference in 2-drug versus 3-drug regimens for grade III or higher infection, pneumonia, and neutropenia across all phases of treatment. The risk of Grade III or higher infection in 2-drug regimens were 1.5% (95% CI = 0.1–2.9%) (2 RCTs, 2 regimens, *n* = 332) [14, 34], while 3-drug regimens were 1.7% (95% CI = 1.3–2.2%) (10 RCTs, 11 regimens, *n* = 2049) [14, 17, 19–22, 26, 33, 35, 36] (Supplementary Figures 6 and 7). For grade III or higher pneumonia, the risk of 2-drug regimens and 3-drug regimens were 1.3 (95% CI = 0.4–2.2%) (2 RCTs, 2 regimens, n = 332) [14, 34] and 1.0 (95% CI = 0.8–1.3%), respectively (10 RCTs, 11 regimens, *n* = 2548) [14, 17, 19, 20, 23, 26, 30, 31, 33, 35] (Supplementary Figures 8 and 9). The risk of Grade III or higher neutropenia in 2-drug regimens was 2.6 (95% CI = -1.9–7.2%) (2 RCTs, 2 regimens, n = 332) [14, 34] and 2.5 (95% CI = 2.0–3.1%) in 3-drug regimens (15 RCTs, 16 regimens, *n* = 3888) [14, 17, 19, 20, 23–26, 30, 31, 33, 35, 36, 38, 39] (Supplementary Figures 10 and 11).

### Use of prophylactic antibiotics in RCTs

Only three RCTs noted whether use of prophylactic antibiotics was mandated (Supplementary Table 4) [24, 27, 43]. Among these studies, name of antibiotics given and number of patients receiving antibiotics were not reported. Only 10 RCTs reported on death from infection [17, 20, 21, 25–28, 35, 37, 43].

### Sensitivity analysis

Omitting single studies successively showed no study had a significant influence on the overall results (Supplementary Figure 12).

## Discussion

Our study is the first study to quantify monthly risk for infection, pneumonia, and neutropenia in various regimens across all phases of treatment in patients with multiple myeloma on clinical trials. We demonstrate that rates of grade III or higher infection, pneumonia, and neutropenia are clinically significant across frontline and RRMM setting. Compared to those in the frontline and RRMM, the complications of infection, pneumonia, and neutropenia in the maintenance setting were low, but not negligible. Our study also indicates that three-drug regimens are no more likely than two-drug regimens to have an increased risk of Grade III or higher infection, implying that multiple patient/host factors (and not just the drugs) play a major part in the causation of infection. Our study thus questions whether there is indeed cumulative toxicity as pertains to infection when additional classes of drugs are added. This merits further study with patient-level information in future work. The importance of host factors is also evident in the frontline setting when using the same drug, VRD, in two different patient populations. VRD was associated with a monthly incidence of grade III or higher infection of 2.6% (2.4–2.8%, I2 = 98.1%) [21] in non-transplant patients compared to 1.5% (1.4–1.6%, I2 = 98.1%) in transplant eligible patients [17]. The difference in infection risks highlights the importance of host factors when assessing infection risks in these patients.

The safety profiles for MM regimens have improved over time, as evidenced by pomalidomide/dexamethasone containing regimens in which the highest risk of Grade III or higher infection per month was seen in the earlier studies (Richardson et al. [45] and MM-003 [46]), with the risk subsequently decreased in later studies (3.5% with bortezomib/pomalidomide/dexamethasone [27], 2.4% for isatuximab, pomalidomide/dexamethasone [35], and 1.6% with elotuzumab/pomalidomide/dexamethasone [29]). As pomalidomide moved from a heavily pre-treated patient population in the earliest aforementioned studies, to earlier in the disease course in more contemporary studies, the risk of Grade III or higher infection decreased significantly. It should be noted that the decreased dose of dexamethasone in contemporary regimens is also likely a contributing factor in the decrease in infections in more modern regimens, as low-dose dexamethasone was associated with better short-term overall survival and lower toxicity when compared to high-dose dexamethasone [47].

Our work provides numerical data to clinicians and patients of the risks of infections, pneumonia, and neutropenia associated with treatment regimens in clinical trials. While extrapolating these results to patients in clinic, it is important to consider differences in baseline characteristics between clinical trial patients and those seen in routine clinical practice, who are often likely to be older, have more comorbidities and potentially be at a higher risk of complications of treatment. Furthermore, the monitoring of infection for routine patients outside of clinical trials may not be as vigorous. Given the risk of infection in trials in not only the newly diagnosed setting, but also the relapsed/refractory setting, consideration must be made for antibiotic prophylaxis strategies in these patients, and trials designing such strategies are needed.

Unfortunately, uniform use of prophylactic antibiotics in clinical trials with MM has historically been lacking [48]. In our analysis, the reporting of whether or not prophylactic antibiotic use was done was inconsistent and sparse and hence could not be ascertained. The use of prophylactic antibiotics has proven to be beneficial in a multicenter randomized trial of levofloxacin prophylaxis for 12 weeks at the start of therapy for newly diagnosed myeloma compared to placebo, owing to reduced febrile episodes and deaths without increasing health-care associated infections [49]. In our study, we did see that because the infection risk did not considerably change between frontline and RRMM, the use of prophylactic antibiotics should be considered in both the frontline and RRMM setting, and this should be an area of future investigation. Subsequent trials should look at the value of antibiotic prophylaxis, as well as the downstream implications such as changes in microbial resistance patterns in the community. As we found that reporting of use antibiotic prophylaxis has historically been poor, ongoing and future trials should clearly report this information.

There are limitations to our study. As most trials use regimens in combination, the exact contribution of each treatment class to the risk of infection, pneumonia, and neutropenia is unknown, and an analysis of the risk of infection per treatment class was not performed. The methodology of our study cannot fully account for other factors on an individual patient level such as prior lines of treatment and other differences in patient characteristics among these clinical trials. Indeed the risk of infection reflects patient specific factors in addition to the toxicity of the drug, and thus direct comparisons must be taken with this caveat. Our search was limited to randomized trials, hence non-randomized study data on regimens currently used such as selinexor-dexamethasone [50] were not included here. Furthermore, unpublished regimens could also not be captured through our search strategy. Studies not clearly mentioning the duration of treatment or our primary outcomes were not included for analysis, decreasing the number of studies evaluated. The marked heterogeneity amongst the patients enrolled on these trials also raises the need for caution before using information for clinical application. Furthermore, our study did not analyze individual types/areas of infection other than pneumonia; Although it is a well-known fact that the patterns of infection vary with class of drugs, such as respiratory infections with the use of anti-CD38 therapy, herpes zoster/herpes simplex infections with PIs, and *Pneumocystis jirovecii* with steroids [1]. We also could not account for when in the course of treatment these infections occurred, or the exact organism that was causing these infections, due to lack of reporting of these variables in the studies analyzed. As our study includes only trials before the onset of COVID-19, our data cannot be used to ascertain the risk of COVID-19 with these regimens, although the data is still of relevance in the COVID era given the well-known complications of neutropenia as they pertain to secondary bacterial infections [51]. There was high statistical heterogeneity in our study as evidenced by the high I2 values, owing to the inclusion of various studies with varying sample sizes and patient populations. This persisted despite sensitivity testing (leave one out analysis).

Prior to our study, there were meta-analyses that studied the risk of infection in MM in specific subsets of patients, however these looked specifically at certain classes of drugs, and not comprehensively at all classes and all phases of treatment [8, 52]. To the best of our knowledge, our study is the first to provide comprehensive quantitative estimates of monthly incidence of events by accounting for duration of treatment, although future work is needed looking at patient level data to account comprehensively for all risk factors.

In summary, our study demonstrates a significant risk of infection in patients treated with various regimens for MM, even in the era of contemporary novel treatments. A transition from chemotherapeutic agents to novel agents has resulted in a decrease in incidence of severe neutropenia, but the incidence of severe infection and pneumonia persists. Infection, pneumonia, and neutropenia remain a risk in frontline, maintenance, and relapsed/refractory setting. Further studies are needed to determine the value of antimicrobial prophylaxis – not only antibacterial, but also antiviral and antifungal medications- in a broader myeloma patient population, as well as other approaches that will further mitigate the morbidity and mortality related to infection in this vulnerable patient population.

## Supplementary Information

**Additional file 1.**

## Data Availability

All data generated or analyzed during this study are included in this published article [and its supplementary information files].

## Abbreviations

MM: Multiple myeloma
IMiDs: Immunomodulators
PI: Protease inhibitors
RCTs: Randomized clinical trials
PRISMA: Preferred Reporting Items for Systemic Reviews and Meta-Analyses
CI: Confidence intervals
VRD: Bortezomib/lenalidomide/dexamethasone
DRd: Daratumumab/lenalidomide/dexamethasone
Vd: Bortezomib/dexamethasone
RRMM: Relapsed/refractory MM
DVd: Daratumumab/bortezomib/dexamethasone
