# Supplementary material for: Infection risks in multiple myeloma: a systematic review and meta-analysis of randomized trials from 2015 to 2019
Source: BMC Cancer. 2021 Jun 26;21:730. doi: 10.1186/s12885-021-08451-x (PMC8233183; doi:10.1186/s12885-021-08451-x)
Supplement: Supplementary file 1 — Additional file 1. [file 12885_2021_8451_MOESM1_ESM.docx]

**Supplementary Figures and Tables**

**Infection Risks in Multiple Myeloma: A systematic review and meta-analysis of randomized trials from 2015-2019**

**Nicole Balmaceda, Muhammad Aziz, Viveksandeep Thoguluva Chandrasekar, Brian McClune, Suman Kambhampati, Leyla Shune, Al-Ola Abdallah, Faiz Anwer, Aneela Majeed, Muzaffar Qazilbash, Siddhartha Ganguly, Joseph McGuirk, Ghulam Rehman Mohyuddin**

*Supplementary Figure 1: Incidence of Grade III or higher pneumonia in frontline myeloma trials*


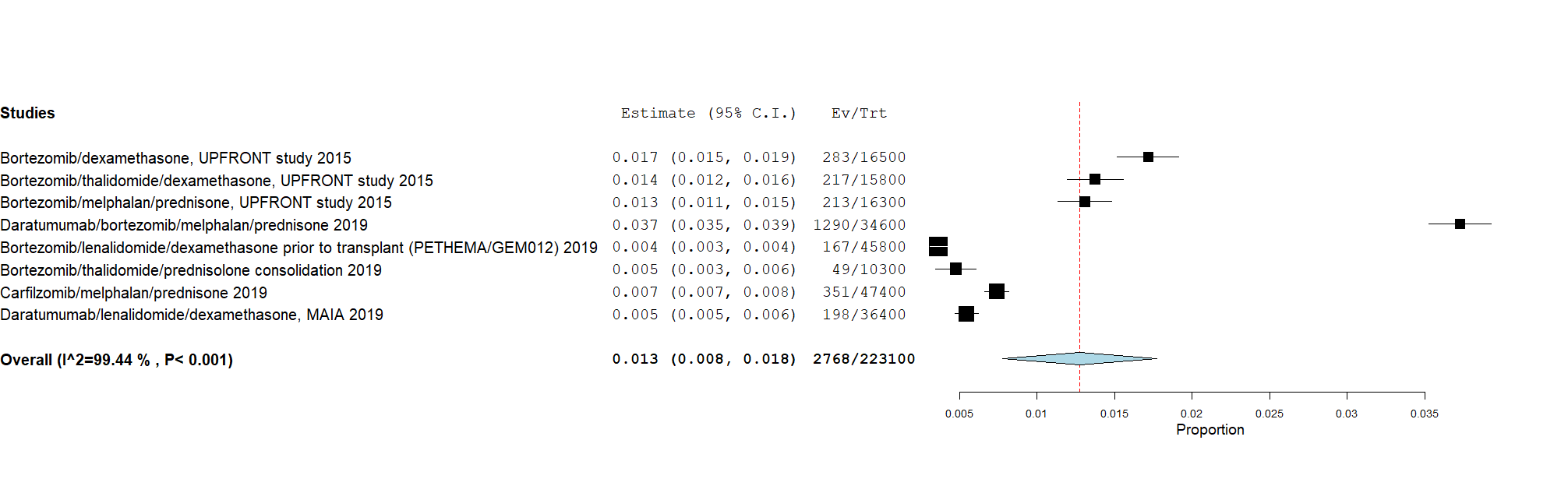


*Supplementary Figure 2: Incidence of Grade III or higher neutropenia in frontline myeloma trials*


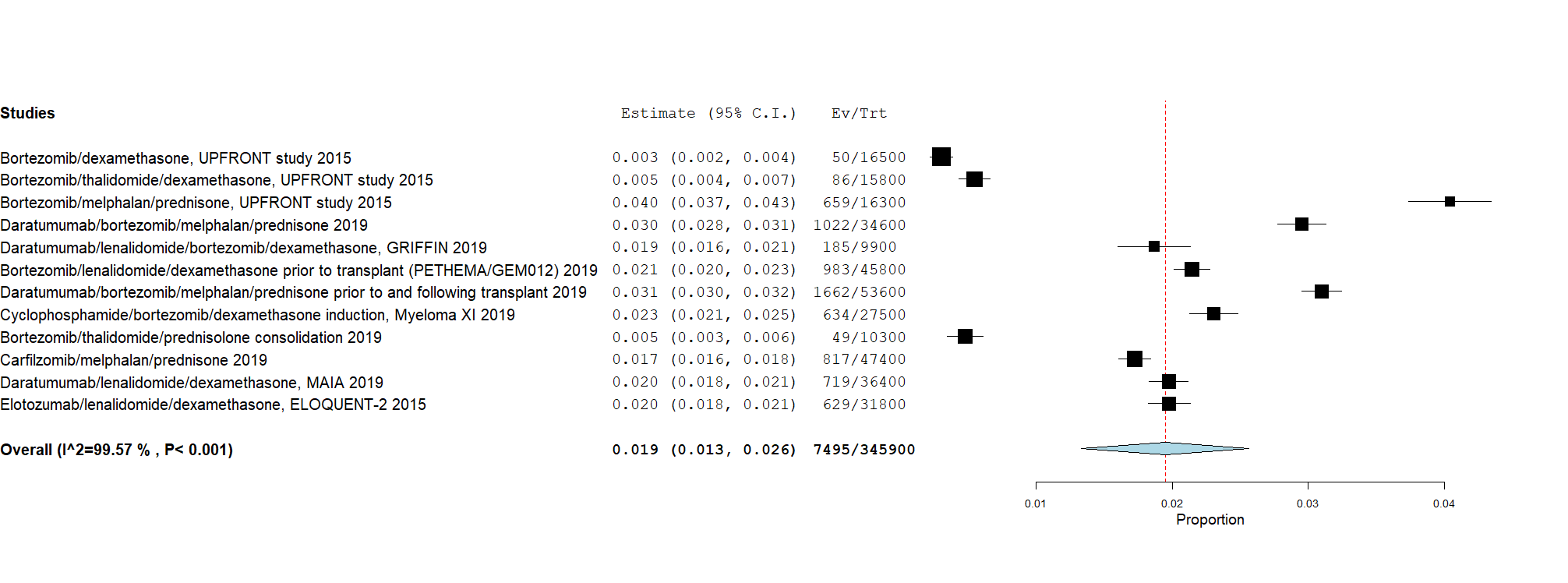


*Supplementary Figure 3: Incidence of Grade III or higher pneumonia in relapsed/refractory myeloma trials*


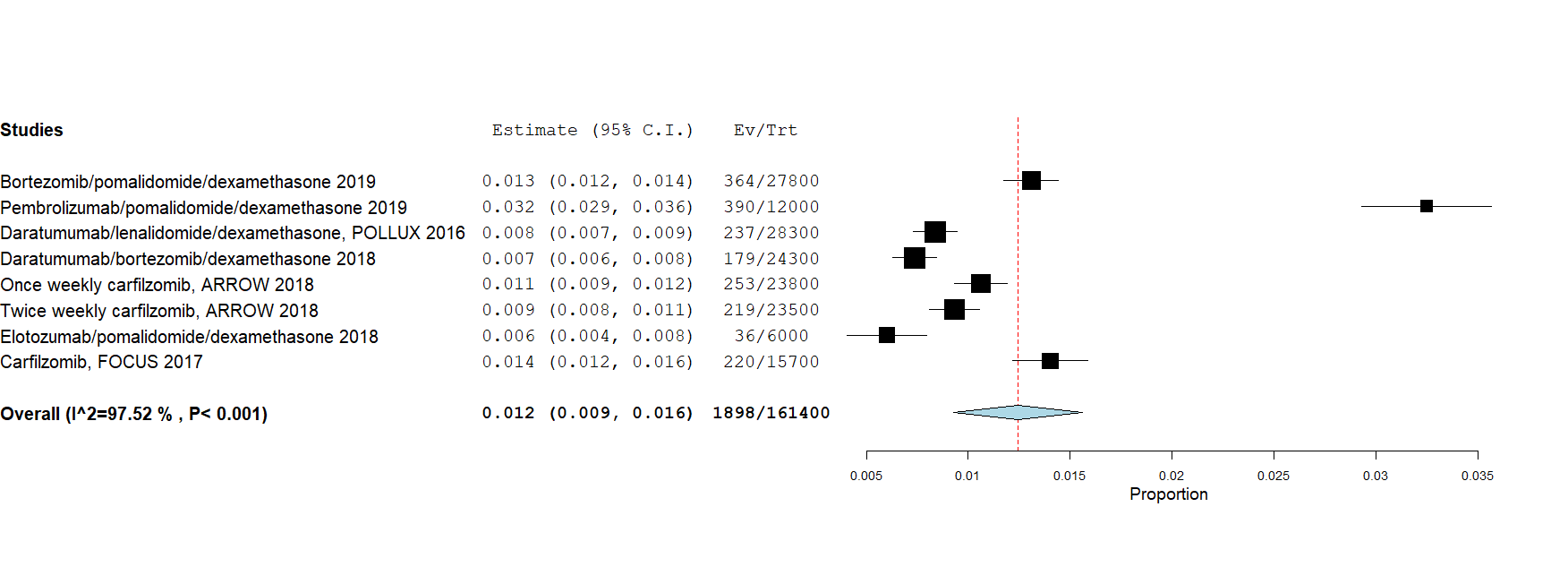


*Supplementary Figure 4: Incidence of Grade III or higher neutropenia in relapsed/refractory myeloma trials*


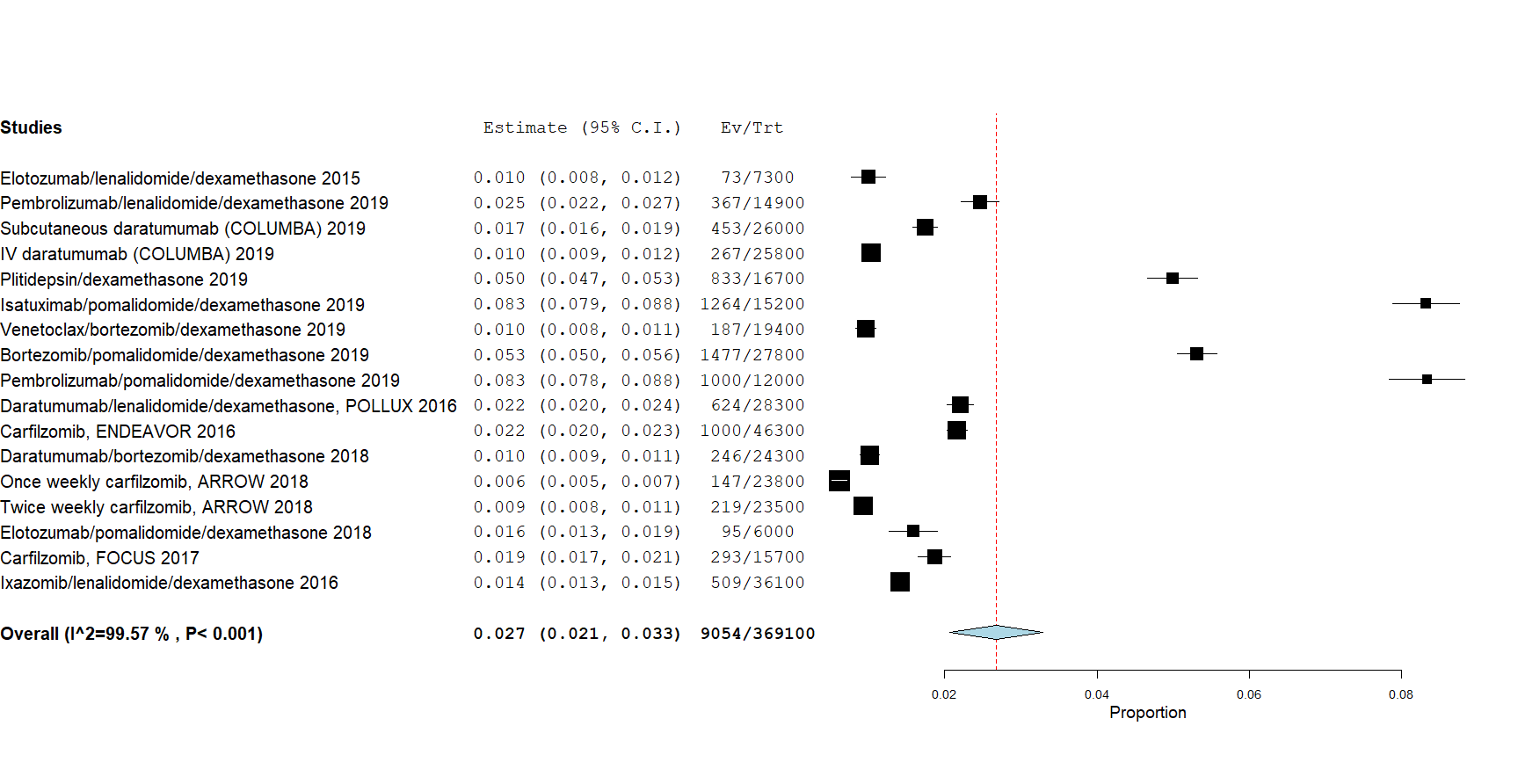


*Supplementary Figure 5: Incidence of Grade III or higher neutropenia in myeloma maintenance trials*


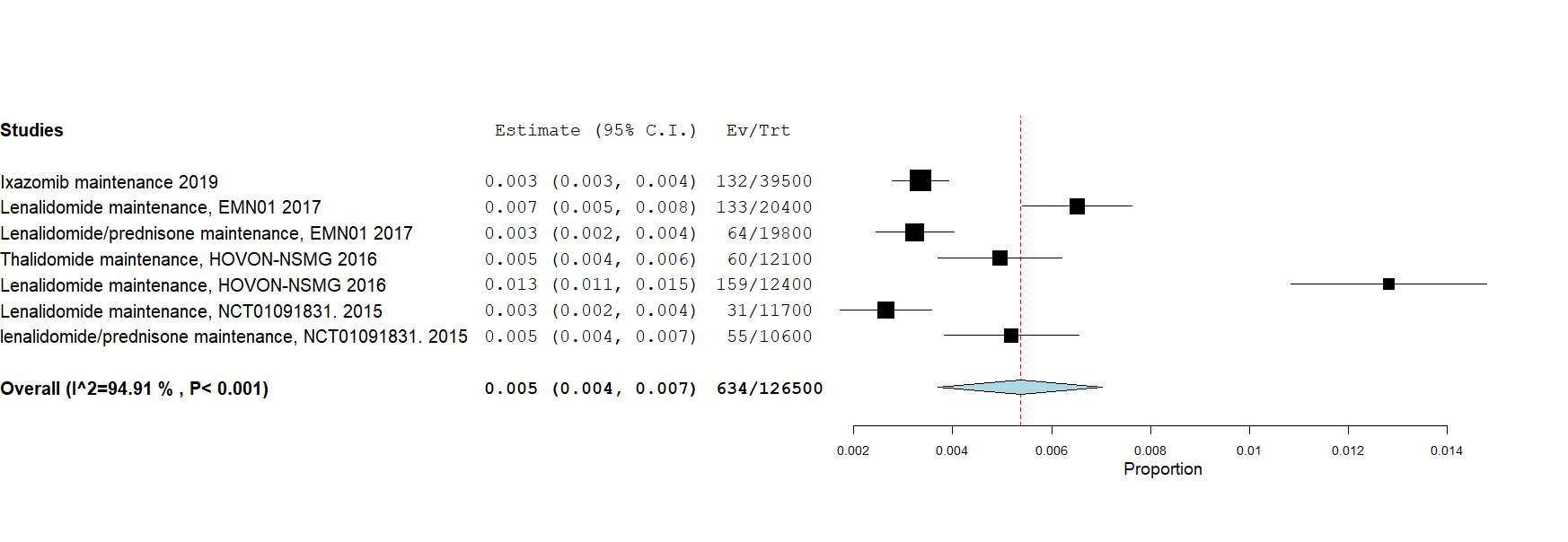


*Supplementary Figure 6: Incidence of Grade III or higher infection in myeloma trials with 2-drug regimens*

*
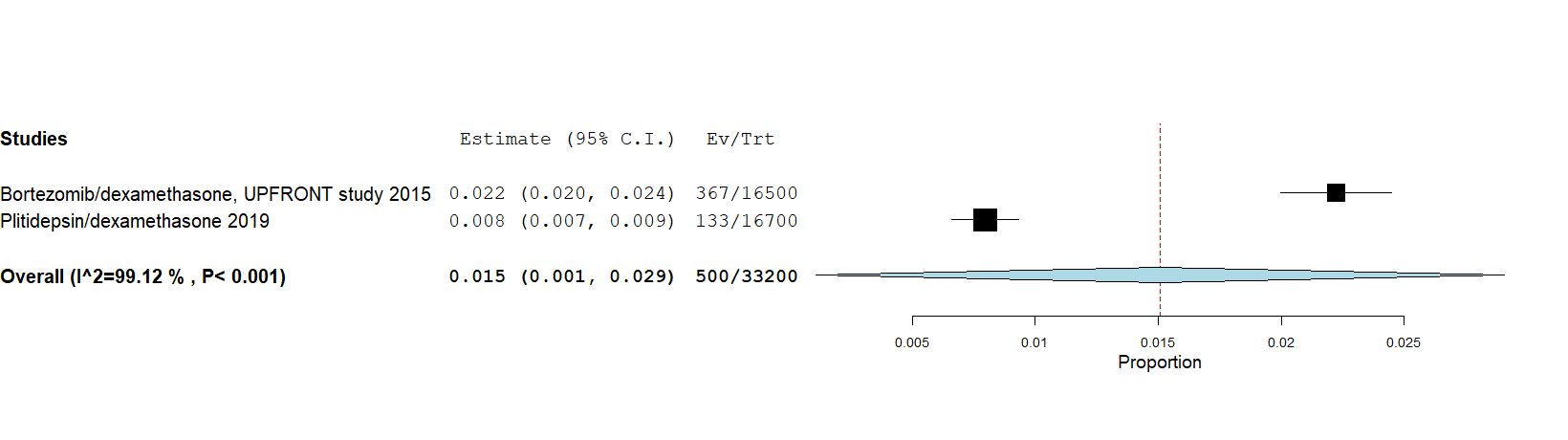
*

*Supplementary Figure 7: Incidence of Grade III or higher infection in myeloma trials with 3-drug regimens*


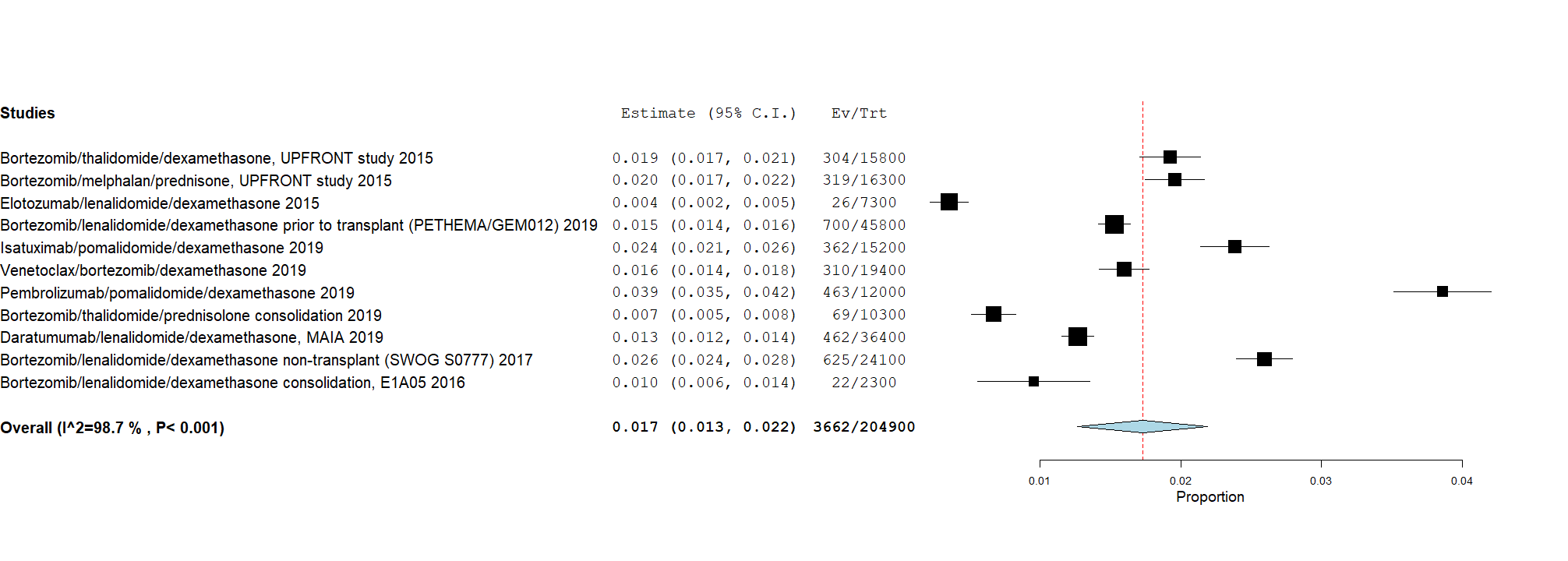


*Supplementary Figure 8: Incidence of Grade III or higher pneumonia in myeloma trials with 2-drug regimens*


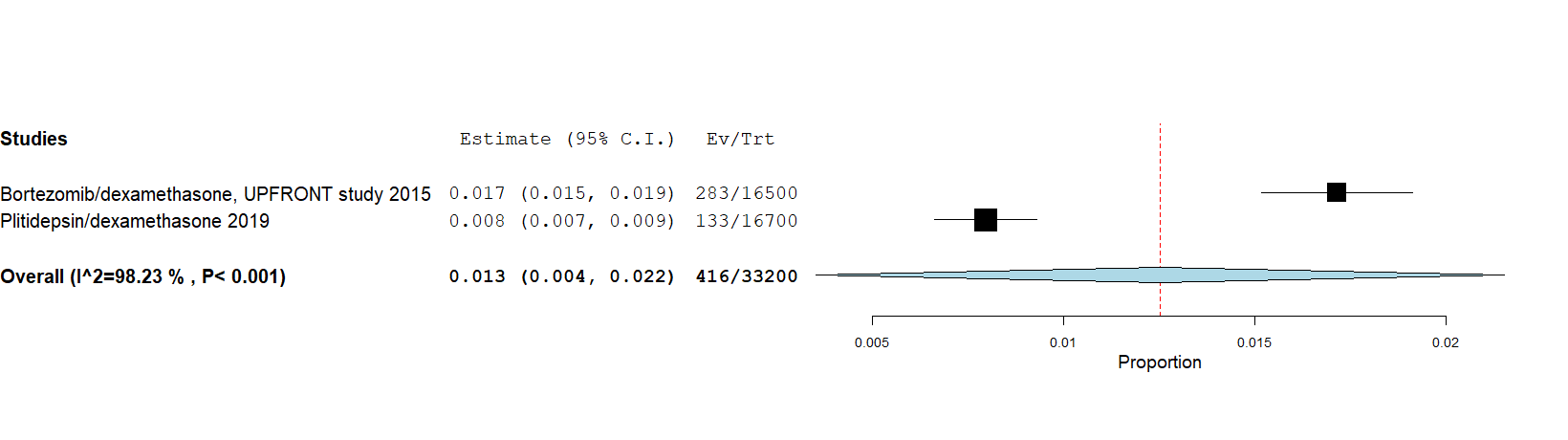


*Supplementary Figure 9: Incidence of Grade III or higher pneumonia in myeloma trials with 3-drug regimens*


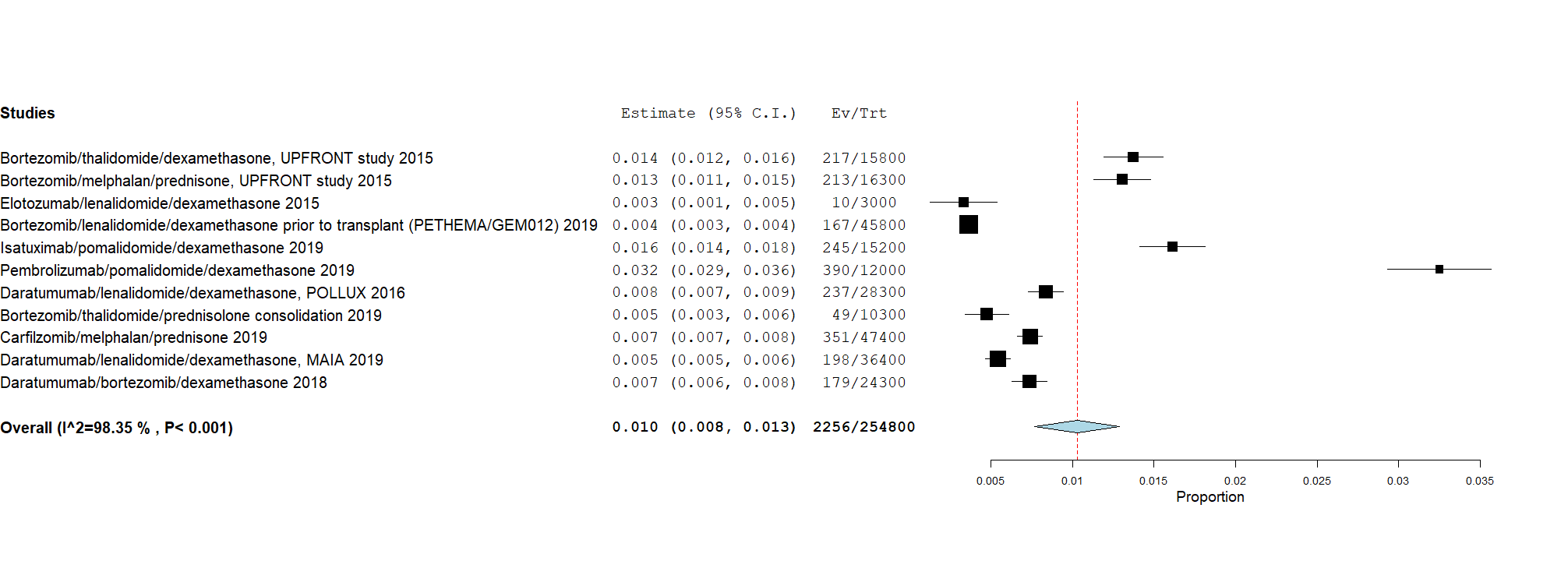


*Supplementary Figure 10: Incidence of Grade III or higher neutropenia in myeloma trials with 2-drug regimens*

*
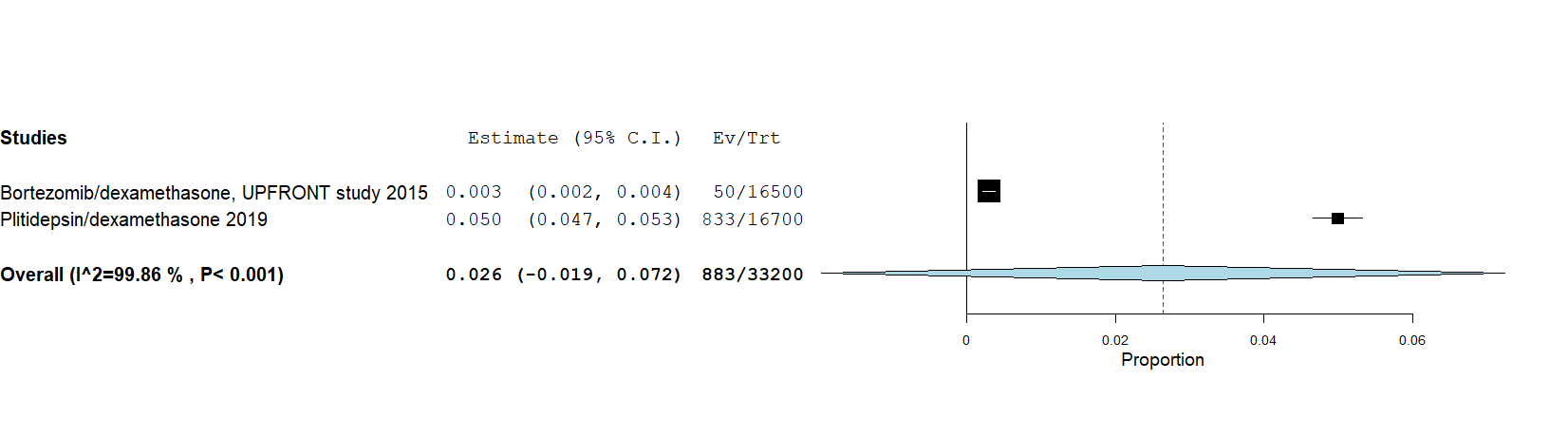
*

*Supplementary Figure 11: Incidence of Grade III or higher neutropenia in myeloma trials with 3-drug regimens*

*
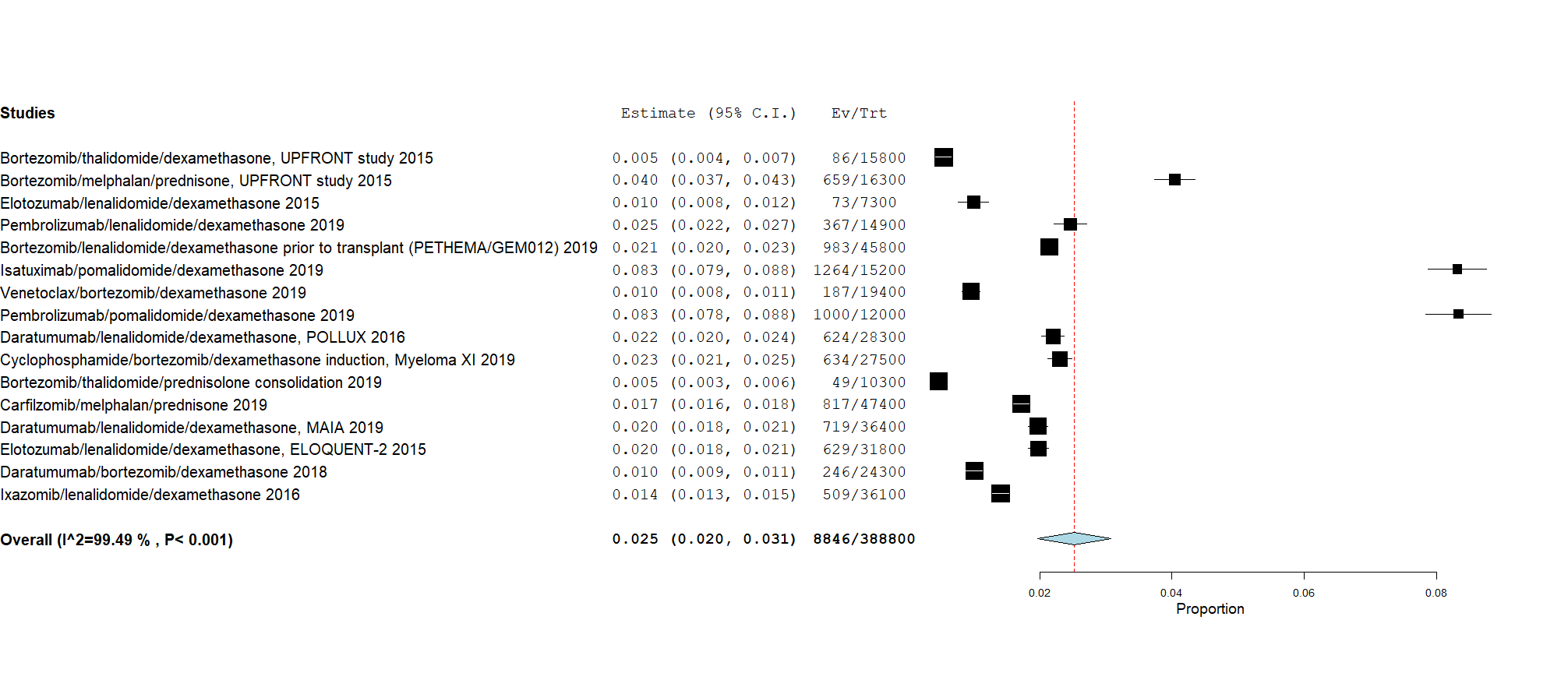
*

*Supplementary Figure 12: Leave one out plot examining the influence of individual studies shows no significant change in pooled grade III or higher infection rate per months with exclusion of any one study*

*Supplementary Table 1: Example search strategy*

| S. No. | Query | Studies |
| --- | --- | --- |
| 1. | myeloma OR myelomas OR myelomatos* OR 'kahler disease' OR 'myeloma'/exp | 108931 |
| 2. | (myeloma OR myelomas OR myelomatos* OR 'kahler disease' OR 'myeloma'/exp) AND [2015-2020]/py | 74264 |
| 3. | #2 AND 'human'/de | 66345 |
| 4. | #2 AND 'human'/de NOT ('conference review'/it OR 'editorial'/it OR 'review'/it OR 'short survey'/it) | 55869 |
| 5. | 'phase 3 clinical trial'/exp | 44510 |
| 6. | 'crossover procedure':de OR 'double-blind procedure':de OR 'randomized controlled trial':de OR 'single-blind procedure':de OR random*:de,ab,ti OR factorial*:de,ab,ti OR crossover*:de,ab,ti OR ((cross NEXT/1 over*):de,ab,ti) OR placebo*:de,ab,ti OR ((doubl* NEAR/1 blind*):de,ab,ti) OR ((singl* NEAR/1 blind*):de,ab,ti) OR assign*:de,ab,ti OR allocat*:de,ab,ti OR volunteer*:de,ab,ti | 2527911 |
| 7. | #5 AND #6 | 32676 |
| 8. | #4 AND #7 | 743 |
| 9. | 'phase 3 clinical trial (topic)'/exp | 36043 |
| 10. | #6 AND #9 | 22280 |
| 11. | #4 AND #10 | 354 |
| 12. | 'kidney disease'/exp/mj OR 'kidney function'/exp/mj OR kidney*:ti OR renal:ti | 784026 |
| 13. | #11 AND #12 | 5 |
| 14. | #8 OR #13 | 748 |

*Supplementary Table 2: Characteristics of included studies (R/R= relapsed/refractory, ND= newly diagnosed, M=maintenance)*

| **Author (Year)** | **Trial Name** | **Study Phase** | **Phase Treatment** | **Regimen** | **No. Patients** | **Median Age (Years)** | **Median Duration of Treatment (Months)** |
| --- | --- | --- | --- | --- | --- | --- | --- |
| Niesvizky  (2015) [14] | UPFRONT | 3 | ND | Bortezomib/dexamethasone | 165 | 74.5 | 6 |
|  |  |  |  | Bortezomib/thalidomide/dexamethasone | 158 | 73 | 4.6 |
|  |  |  |  | Bortezomib/melphalan/prednisone | 163 | 72 | 4.7 |
| Richardson (2015)[26] |  | 2 | RR | Elotuzumab/lenalidomide/dexamethasone | 73 | 62 | 19.1 |
| Mateos (2019) [15] | ALCYONE | 3 | ND | Daratumumab/bortezomib/melphalan/prednisone | 346 | 71 | 13.5 |
| Voorhees (2019) [16] | GRIFFIN | 2 | ND | Daratumumab/lenalidomide/bortezomib/dexamethasone | 99 | 59 | 22.1 |
| Usmani (2019)[38] | KEYNOTE-185 | 3 | RR | Pembrolizumab/lenalidomide/dexamethasone | 149 | 74 | 4.4 |
| Mateos (2019)  [40] | COLUMBA | 3 | RR | Subcutaneous daratumumab | 260 | 65 | 7.5 |
|  |  |  |  | IV daratumumab | 258 | 68 | 7.5 |
| Spicka (2019)[27] | ADMYRE | 3 | RR | Plitidepsin/dexamethasone | 167 | 64 | 3 |
| Rosinol (2019)[17] | PETHEMA/GEM2012 | 3 | ND | Bortezomib/lenalidomide/dexamethasone prior to transplant | 458 | 58 | 6 |
| Attal (2019) [28] | ICARIA-MM | 3 | RR | Isatuximab/pomalidomide/dexamethasone | 152 | 68 | 10.2 |
| Morgan (2019)[41] | TOURMALINE-MM3 study | 3 | M | Ixazomib maintenance | 395 | 58 | 15.2 |
| Moreau (2019) [29] | BELLINI | 3 | RR | Venetoclax/bortezomib/dexamethasone | 194 | 66 | 18.7 |
| Moreau (2019) [18] | CASSIOPEIA | 3 | ND | Daratumumab/bortezomib/melphalan/prednisone prior to and following transplant | 536 | 59 | 8.9 |
| Richardson (2019)[30] | OPTISIMISMM | 3 | RR | Bortezomib/pomalidomide/dexamethasone | 278 | 67 | 8.8 |
| Mateos (2019) [31] | KEYNOTE-183 | 3 | RR | Pembrolizumab/pomalidomide/dexamethasone | 120 | 65 | 4.1 |
| Dimopoulos (2018)[35] | POLLUX | 3 | RR | Daratumumab/lenalidomide/dexamethasone | 283 | 65 | 24.5 |
| Jackson (2019)[24] | Myeloma XI | 3 | ND | Cyclophosphamide/bortezomib/dexamethasone induction | 275 | 66 | 2.8 |
| Horvath (2019)[19] | VCAT | 3 | ND | Bortezomib/thalidomide/prednisolone consolidation | 103 | 58 | 10.2 |
| Facon (2019) [23] | CLARION | 3 | ND | Carfilzomib/melphalan/prednisone | 474 | 72 | 13.1 |
| Facon (2019) [20] | MAIA | 3 | ND | Daratumumab/lenalidomide/dexamethasone | 364 | 73 | 25.3 |
| Lonial (2015) [25] | ELOQUENT-2 | 3 | RR | Elotuzumab/lenalidomide/dexamethasone | 318 | 67 | 17 |
| Dimopoulos (2016)[32] | ENDEAVOR | 3 | RR | Carfilzomib | 463 | 65 | 10 |
| Spencer (2018) [36] | CASTOR | 3 | RR | Daratumumab/bortezomib/dexamethasone | 243 | 64 | 13.4 |
| Moreau (2018)[33] | A.R.R.O.W. | 3 | RR | Once weekly carfilzomib | 238 | 66 | 9.5 |
|  |  |  |  | Twice weekly carfilzomib | 235 | 66 | 7.3 |
| Dimopoulos  (2018) [34] | ELOQUENT-3 | 2 | RR | Elotuzumab/pomalidomide/dexamethasone | 60 | 69 | 8.4 |
| Hajek (2016) [37] | FOCUS | 3 | RR | Carfilzomib | 157 | 63 | 4.1 |
| Durie (2017)[21] | SWOG S0777 | 3 | ND | Bortezomib/lenalidomide/dexamethasone non-transplant | 242 | 63 | 5.6 |
| Bringhen (2019)[42] | EMN01 | 3 | M | Lenalidomide maintenance | 204 | 73 | 32.4 |
|  |  |  |  | Lenalidomide/prednisone maintenance | 198 | 73 | 29.8 |
| Zweegman (2016) [43] | HOVON-NSMG | 3 | M | Thalidomide maintenance | 121 | 72 | 5 |
|  |  |  |  | Lenalidomide maintenance | 124 | 73 | 17 |
| Moreau (2016)[39] |  | 3 | RR | Ixazomib/lenalidomide/dexamethasone | 361 | 66 | 15.9 |
| Jacobus (2016)[22] | E1A05 | 3 | NF | Bortezomib/lenalidomide/dexamethasone consolidation, | 23 |  | 4.55 |
| Gay (2015)[44] |  | 3 | M | Lenalidomide maintenance | 117 | 57 | 28.9 |
|  |  |  |  | lenalidomide/prednisone maintenance | 106 | 56 | 25.3 |

*Supplementary Table 3: Risk of bias for included studies*

| **Author (Year)** | **Selection bias, as pertains to random sequence generation** | **Selection bias related to allocation concealment** | **Performance bias due to non-blinding of participants and investigators** | **Detection bias due to non-blinding of outcome assessment** | **Attrition bias due to incomplete outcome data** | **Reporting bias due to selective reporting** |
| --- | --- | --- | --- | --- | --- | --- |
| Niesvizky (2015) | NO | NO | YES | NO | NO | NO |
| Richardson (2015) | NO | NO | YES | NO | NO | NO |
| Mateos (2019) | NO | NO | YES | NO | NO | NO |
| Voorhees (2019) | NO | NO | YES | NO | NO | NO |
| Usmani (2019) | NO | NO | YES | NO | NO | NO |
| Mateos (2019) | NO | NO | YES | NO | NO | NO |
| Spicka (2019) | NO | NO | YES | NO | NO | NO |
| Rosinol (2019) | NO | NO | YES | NO | NO | NO |
| Attal (2019) | NO | NO | YES | NO | NO | NO |
| Morgan (2019) | NO | NO | NO | NO | NO | NO |
| Moreau (2019) | NO | NO | NO | NO | NO | NO |
| Moreau (2019) | NO | NO | YES | NO | NO | NO |

*Supplementary Table 4: Table S4: Antibiotics use of included studies*

| **Author (Year)** | **Regimen** | **No. Patients** | **Antibiotic Prophlaxis Mandated** | **No. Patients Recived Antibiotics** | **Name Antibiotics Given** | **No. Patients Died from Infection** |
| --- | --- | --- | --- | --- | --- | --- |
| Niesvizky (2015) | Bortezomib/dexamethasone | 165 | NA | NA | NA |  |
|  | Bortezomib/thalidomide/dexamethasone | 158 | NA | NA | NA |  |
|  | Bortezomib/melphalan/prednisone | 163 | NA | NA | NA |  |
| Richardson (2015) | Elotuzumab/lenalidomide/dexamethasone | 73 | NA | NA | NA |  |
| Mateos (2019) | Daratumumab/bortezomib/melphalan/prednisone | 346 | NA | NA | NA |  |
| Voorhees (2019) | Daratumumab/lenalidomide/bortezomib/dexamethasone | 99 | NA | NA | NA |  |
| Usmani (2019) | Pembrolizumab/lenalidomide/dexamethasone | 149 | NA | NA | NA |  |
| Mateos (2019) | Subcutaneous daratumumab | 260 | NA | NA | NA |  |
|  | IV daratumumab | 258 | NA | NA | NA |  |
| Spicka (2019) | Plitidepsin/dexamethasone | 167 | NA | NA | NA |  |
| Rosinol (2019) | Bortezomib/lenalidomide/dexamethasone prior to transplant | 458 | NA | NA | NA | 4 |
| Attal (2019) | Isatuximab/pomalidomide/dexamethasone | 152 | NA | NA | NA | 1 |
| Morgan (2019) | Ixazomib maintenance | 395 | NA | NA | NA |  |
| Moreau (2019) | Venetoclax/bortezomib/dexamethasone | 194 | NA | NA | NA |  |
| Moreau (2019) | Daratumumab/bortezomib/melphalan/prednisone prior to and following transplant | 536 | NA | NA | NA |  |
| Richardson (2019) | Bortezomib/pomalidomide/dexamethasone | 278 | Permitted | NA | NA | 5 |
| Mateos (2019) | Pembrolizumab/pomalidomide/dexamethasone | 120 | NA | NA | NA | 5 |
| Dimopoulos (2018) | Daratumumab/lenalidomide/dexamethasone | 283 | NA | NA | NA |  |
| Jackson (2019) | Cyclophosphamide/bortezomib/dexamethasone induction | 275 | Permitted | NA | NA |  |
| Horvath (2019) | Bortezomib/thalidomide/prednisolone consolidation | 103 | NA | NA | NA |  |
| Facon (2019) | Carfilzomib/melphalan/prednisone | 474 | NA | NA | NA |  |
| Facon (2019) | Daratumumab/lenalidomide/dexamethasone | 364 | NA | NA | NA | 18 |
| Lonial (2015) | Elotuzumab/lenalidomide/dexamethasone | 318 | NA | NA | NA | 2 |
| Dimopoulos (2016) | Carfilzomib | 463 | NA | NA | NA | 6 |
| Spencer (2018) | Daratumumab/bortezomib/dexamethasone | 243 | NA | NA | NA |  |
| Moreau (2018) | Once weekly carfilzomib | 238 | NA | NA | NA | 1 |
|  | Twice weekly carfilzomib | 235 | NA | NA | NA |  |
| Dimopoulos (2018) | Elotuzumab/pomalidomide/dexamethasone | 60 | NA | NA | NA |  |
| Hajek (2016) | Carfilzomib | 157 | NA | NA | NA |  |
| Durie (2017) | Bortezomib/lenalidomide/dexamethasone non-transplant | 242 | NA | NA | NA | 1 |
| Bringhen (2019) | Lenalidomide maintenance | 204 | NA | NA | NA |  |
|  | Lenalidomide/prednisone maintenance | 198 | NA | NA | NA |  |
| Zweegman (2016) | Thalidomide maintenance | 121 | Permitted | NA | NA | 6 |
|  | Lenalidomide maintenance | 124 | Permitted | NA | NA | 8 |
| Moreau (2016) | Ixazomib/lenalidomide/dexamethasone | 361 | NA | NA | NA |  |
| Jacobus (2016) | Bortezomib/lenalidomide/dexamethasone consolidation, | 23 | NA | NA | NA |  |
| Gay (2015) | Lenalidomide maintenance | 117 | NA | NA | NA |  |
|  | lenalidomide/prednisone maintenance | 106 | NA | NA | NA |  |
